# Liver cirrhosis may increase the incidence of delayed bleeding and mortality after endoscopic retrograde cholangiopancreatography for common bile duct stones

**DOI:** 10.3389/fmed.2025.1619929

**Published:** 2025-07-24

**Authors:** Linzhen Li, Di Wang, Xiaoping Niu

**Affiliations:** ^1^Department of Gastroenterology, First Affiliated Hospital of Wannan Medical College, Wuhu, Anhui, China; ^2^Department of Gastroenterology, The Affiliated Hospital of Xuzhou Medical University, Xuzhou, Jiangsu, China

**Keywords:** choledocholithiasis, cirrhosis, ERCP, delayed hemorrhage, mortality

## Abstract

**Background and purpose:**

Choledocholithiasis is a common disease. However, the results of studies on the complications of choledocholithiasis with cirrhosis by endoscopic retrograde cholangiopancreatography (ERCP) are inconsistent. Therefore, the purpose of this prospective study is to compare the incidence of postoperative complications of ERCP between choledocholithiasis with cirrhosis and those without cirrhosis.

**Patients and methods:**

A total of 259 choledocholithiasis patients were include in this study. According to whether the choledocholithiasis patients were complicated with cirrhosis, they were divided into cirrhosis group and non-cirrhosis group. The incidence of post-ERCP pancreatitis (PEP), delayed bleeding, infection, perforation, and mortality were compared between the two groups.

**Results:**

There were 34 choledocholithiasis patients with cirrhosis and 225 patients without cirrhosis. There were statistically significant differences in delayed hemorrhage (*p =* 0.046) and mortality (*p =* 0.017) between the two groups. The rate of delayed hemorrhage and mortality in choledocholithiasis with cirrhosis patients group were significantly higher than those without cirrhosis. There were no statistically significant differences in PEP (*p =* 0.917), postoperative biliary tract infection (*p =* 0.130), stone clearance (*p =* 0.201), and perforation (*p* = 1.000) between the two groups.

**Conclusion:**

The results of this study found that cirrhosis may be associated with the occurrence of ERCP complications. Delayed hemorrhage and mortality after ERCP in choledocholithiasis patients with cirrhosis were significantly higher than those without cirrhosis.

## 1 Introduction

Choledocholithiasis is an acute or chronic calculous disease located in the common bile duct, often occurring at the lower end of the common bile duct. According to their source, they can be divided into primary and secondary choledocholithiasis. The formation of primary gallstones in bile ducts is closely related to biliary tract infection, cholestasis and biliary ascaris. Bile duct stones from the gallbladder, called secondary bile duct stones, cholesterol stones are more common. The prevalence of choledocholithiasis varies from country to country, ranging from 8 to 18% in symptomatic gallstones (1–5). Typical choledocholithiasis will have abdominal pain, chills, high fever and jaundice, even combined with a drop in blood pressure and neuropsychiatric symptoms. During physical examination, yellow skin, sclera, right upper abdominal tenderness, rebound pain, muscle tension can be found. European guidelines recommend that all suitable patients with choledocholithiasis should be treated with lithotomy (6). Choledocholithiasis can be treated with endoscopic retrograde cholangiopancreatography (ERCP). ERCP is the most common method for choledocholithiasis removal according to current guidelines (7, 8). However, ERCP also has associated complications, including post-ERCP pancreatitis (PEP), bleeding, perforation, infection, etc. Among them, PEP is the most common complication of ERCP, with a reported incidence of about 10.2%, and even 14.1% for high-risk groups (9). Bleeding is one of the most serious complications of ERCP, with an incidence of about 0.3 to 2% (10–13).

Cirrhosis is a chronic progressive liver disease that is caused by multiple causes of long-term or repeated liver damage. The common causes are viral hepatitis, fatty liver, alcoholic liver, drug hepatitis and so on, of which chronic hepatitis B is the most common patient. The main clinical manifestations are liver function damage and portal hypertension, and multiple organs are involved. Complications such as upper digestive tract bleeding, hepatic encephalopathy, hepatorenal syndrome, secondary infection and liver cancer may occur in the late stage. Patients with cirrhosis are prone to choledocholithiasis, and cirrhosis with acute cholangitis may have a high mortality rate due to bacterial infection and permanent liver dysfunction caused by acute liver injury and cholestasis. Studies showed that the cirrhotic group had a significantly higher mortality rate than the non-cirrhotic group after ERCP (14, 15). Therefore, the purpose of this study was to compare the postoperative complications of ERCP between choledocholithiasis patients with cirrhosis and choledocholithiasis patients without cirrhosis.

## 2 Methods

### 2.1 Ethical considerations

The research was performed according to the Declaration of Helsinki including participants’ consent. The study was approved by the local Ethics Committee.

### 2.2 Patients and study design

Due to the inconsistent results of studies on postoperative complications after ERCP treatment in choledocholithiasis patients with liver cirrhosis, and most of them being retrospective studies, we conducted a prospective study to evaluate the impact of liver cirrhosis on post-ERCP complications of choledocholithiasis.

This study included choledocholithiasis patients who were admitted to First Affiliated Hospital of Wannan Medical College for ERCP treatment during the period from July 2022 to July 2024. The diagnosis of liver cirrhosis depends on the results of previous or post-hospital imaging and endoscopic examinations that indicate liver cirrhosis. All participants were operated on by the same endoscopic doctor who has over 10 years of rich experience in ERCP. The inclusion criteria were as follows: (1) patients who meet the diagnostic criteria for choledocholithiasis, (2) choledocholithiasis patients received ERCP treatment. The exclusion criteria were as follows: (1) ERCP intubation failed and was transferred to surgery, (2) acute pancreatitis occurred before ERCP.

Gender, age were recorded in the participants when they were first admitted to hospital. Acute pancreatitis, delayed bleeding, infection, perforation and death were followed up closely within a month after ERCP. A diagnosis of acute pancreatitis requires two out of three criteria: (1) abdominal pain, (2) a serum amylase or lipase increased at least threefold, and (3) findings consistent with pancreatitis on cross-sectional abdominal imaging (16, 17). Delayed bleeding refers to bleeding that occurs within a few hours to 1 month after the operation. Perforation includes intraoperative perforation and delayed perforation. Infections include acute cholangitis and duodenoscope-associated infections.

According to whether the choledocholithiasis patients were complicated with cirrhosis, they were divided into cirrhosis group and non-cirrhosis group. The incidence of PEP, delayed bleeding, infection, perforation, and mortality were recorded between the two groups. Then compare the above indexes between the two groups.

### 2.3 Statistical analysis

Descriptive data are expressed in terms of median (interquartile range) or counts and percentages. All numerical variables were tested for normal distribution using Shapiro–Wilk test. Mann–Whitney U test was used for nonparametric tests, and Chi-square test or Fisher’s exact test was used for categorical variables. SPSS 21.0 software was used for statistical analysis. A *p*-value < 0.05 indicated statistical significance.

## 3 Results

### 3.1 Clinical features of all choledocholithiasis patients

A total of 259 choledocholithiasis patients were included. There were 124 males and 135 females. The median (interquartile distance) of age was 69 (58–79). Twenty-eight (10.81%) patients developed postoperative acute pancreatitis. Delayed postoperative hemorrhage occurred in 3 (1.16%) participants. Postoperative biliary tract infection occurred in 5 (1.93%) participants. Postoperative biliary perforation occurred in only 1 (0.39%) participant. In 26 participants, choledocholithiasis could not be completely removed, mostly due to the size of the stones. Two participants died postoperatively, one due to delayed hemorrhage and the other due to severe biliary tract infection. Thirty-four choledocholithiasis patients had cirrhosis and another 225 choledocholithiasis patients did not have cirrhosis (Table 1).

**Table 1:** Analysis of postoperative complications between cirrhosis group and non-cirrhosis group.

Parameters	All participants	Cirrhosis group	Non-cirrhosis group	*p*
		*n* = 34	*n* = 225	
Acute pancreatitis: *n* (%)	28 (10.81%)	3 (8.82%)	25 (11.11%)	0.917
Delayed hemorrhage: *n* (%)	3 (1.16%)	2 (5.88%)	1 (0.44%)	0.046
Postoperative biliary tract infection: *n* (%)	5 (1.93%)	2 (5.88%)	3 (1.33%)	0.130
Perforation of biliary tract or duodenum: *n* (%)	1 (0.39%)	0 (0.00%)	1 (0.44%)	1.000
Residual bile duct stones: *n* (%)	26 (10.04%)	6 (17.65%)	20 (8.89%)	0.201
Postoperative death: *n* (%)	2 (0.77%)	2 (5.88%)	0 (0.00%)	0.017

M, median; QR, Quartile Range.

### 3.2 Analysis of the basic characteristics between cirrhosis group and non-cirrhosis group

There were 22 males and 12 females in choledocholithiasis with cirrhosis group, while 102 males and 123 females in choledocholithiasis without cirrhosis group. The difference was statistically significant (*p* = 0.035). There were also statistically significant differences between the two groups in terms of Child-Pugh score (*p* = 0.028), international normalized ratio (INR) (*P* < 0.001), the presence of ascites (*P* < 0.001), presence of portal hypertension (*P* < 0.001), and stent placement (*p* = 0.016). However, there were no significant statistical differences between the two groups in terms of age (*p* = 0.455), platelets (*p* = 0.053), need for sphincterectomy (*p* = 0.372), diabetes (*p* = 0.754), chronic kidney disease (*p* = 1.000), hypertension (*p* = 0.732), and coronary heart disease (*p* = 0.07) (Table 2).

**Table 2:** Analysis of the basic characteristics between cirrhosis group and non-cirrhosis group.

Parameters	Cirrhosis group	Non-cirrhosis group	*p*
	*n* = 34	*n* = 225	
Sex (M/F)	22/12	102/123	0.035
Age (years): M (QR)	69 (59.75–80.25)	69 (57–79)	0.455
Child-Pugh: *n*	6 (5–7)	5 (5–7)	0.028
Platelets: *n*, *10^9^/L	154 (76–213)	168 (130–216)	0.053
INR: *n*	1.13 (1.05–1.22)	1.04 (0.99–1.11)	<0.001
Presence of ascitis: *n* (%)	11 (32.35%)	8 (3.56%)	<0.001
Presence of portal hypertension: *n* (%)	10 (29.41%)	0 (0%)	<0.001
Need for sphincterectomy: *n* (%)	29 (85.29%)	177 (78.67%)	0.372
Stent placement: *n* (%)	4 (11.76%)	41 (18.22%)	0.016
Diabetes: *n* (%)	4 (11.76%)	21 (10.67%)	0.754
Chronic kidney disease: *n* (%)	1 (2.94%)	6 (2.67%)	1.000
Hypertension: *n* (%)	13 (38.24%)	93 (41.33%)	0.732
Coronary heart disease: *n* (%)	5 (14.71%)	13 (5.78%)	0.07

M, median; QR, Quartile Range; INR, international normalized ratio.

### 3.3 Comparison of postoperative complications of ERCP between choledocholithiasis with cirrhosis group and choledocholithiasis without cirrhosis group

There were significant differences in delayed hemorrhage (*p =* 0.046) and postoperative death (*p =* 0.017) between the two groups. The rate of delayed hemorrhage and mortality in choledocholithiasis with cirrhosis patients group were statistically significantly higher than those without cirrhosis. Two choledocholithiasis with cirrhosis patients died, and none of the choledocholithiasis patients without cirrhosis died. There were no significant differences in age (*p =* 0.455), postoperative acute pancreatitis (*p =* 0.917), postoperative biliary tract infection (*p =* 0.130) and perforation of biliary tract or duodenum (*p =* 1.000) between the two groups. The rate of incomplete removal of choledocholithiasis with cirrhosis patients was higher than that in patients without cirrhosis (17.65% vs. 8.89%), but the difference was not statistically significant (*p =* 0.201) (Table 1).

## 4 Discussion

Choledocholithiasis is a common digestive tract disease. Complications of choledocholithiasis include acute pancreatitis, cholangitis (18) and others. Therefore, early diagnosis and treatment of choledocholithiasis are very necessary. ERCP is the gold standard for diagnosing choledocholithiasis and is the preferred treatment for choledocholithiasis (19, 20). However, ERCP also has many complications, such as postoperative acute pancreatitis, delayed hemorrhage, perforation, and biliary tract infection. PEP is the most common complication. A study published in 2024 showed that the incidence of PEP in patients with asymptomatic choledocholithiasis was 11.7% (21). A study by Çağatay et al. (22) showed that the incidence of PEP was 7.9%. In our study, PEP also was the most common complication, with an incidence of 10.81%, basically consistent with the above two studies. However, there was no significant difference in the incidence of PEP between the choledocholithiasis with cirrhosis group and the group without cirrhosis (8.82% vs. 11.11%). A retrospective study published in 2022 (23) showed that patients with cirrhosis were at higher risk for PEP, the postoperative acute incidence was as high as 44% in the cirrhosis group. Another study (24) also found the incidence of PEP was higher in the cirrhosis group than non-cirrhosis group. But a study showed that patients with cirrhosis had lower incidence of post-ERCP pancreatitis (8.6% vs. 7%; *p* < 0.0001) (25). This study is not consistent with the above findings. However, a study (26) published in 2014 proved that there was no significant difference in the incidence of PEP between the cirrhotic and non-cirrhotic groups, which is consistent with the results of this study. The reason may be that the patients with postoperative amylase elevation but no abdominal pain or imaging signs were not classified as PEP in this study. Retrospective studies may not be able to do this accurately.

In this study, the delayed hemorrhage rate in the cirrhosis group was significantly higher than that in the non-cirrhosis group (5.88% vs. 0.44%, *p* = 0.046). Previous studies have shown that the incidence of delayed hemorrhage is 0.3–10.9% (10–13, 27, 28). The rate of delayed hemorrhage in the group without cirrhosis was consistent with previous studies, and the rate of hemorrhage in the group with cirrhosis was significantly higher than that in previous studies. Sumant Inamdar et al. (24) found that ERCP-associated bleeding were more common in the cirrhosis group than non-cirrhosis group (2.3% vs. 1.0%). This is consistent with our findings. Analysis of the reason may be that decompensated cirrhosis patients with reduced liver function, so that the liver synthesis of clotting factors decreased, which function weakened, more prone to postoperative delayed bleeding. In this study, the INR of the liver cirrhosis patient group was significantly higher than that of the non-liver cirrhosis group, confirming this speculation. In addition, patients with cirrhosis are often complicated with hypersplenism, resulting in thrombocytopenia, and the function of platelets is to participate in hemostasis, and thrombocytopenia leads to weakened hemostatic function. In this study, the platelet count in the liver cirrhosis group was lower than that in the non-liver cirrhosis group. Although there was no significant statistical difference, the *p* value was 0.053, which was very close to 0.05. This does not rule out the possibility that the lack of significant difference was due to the small sample size. There is also increased capillary fragility in patients with cirrhosis, coupled with the lack of vitamin C in patients, and cannot make good use of vitamin potassium, anticoagulant substances in the blood increase, will increase bleeding. In this study, there were no significant differences between the group with liver cirrhosis and the group without liver cirrhosis in terms of diabetes, hypertension, coronary heart disease, and chronic kidney disease. This excluded the influence factors of underlying diseases on delayed bleeding. Therefore, ERCP treatment for choledocholithiasis with cirrhosis patients requires sufficient hemostasis during the operation, and close attention should be paid to the occurrence of delayed hemorrhage after surgery, and measures should be actively taken to deal with delayed hemorrhage.

The two choledocholithiasis patients who died in this study had cirrhosis and were both elderly. One of the patients died of delayed postoperative hemorrhage and the other died of septic shock due to severe postoperative biliary tract infection. These results showed that elderly choledocholithiasis patients with cirrhosis may have higher risk of death after ERCP. The results of this study are consistent with previous studies (14). As to whether there are significant differences in biliary tract infection, perforation and stone clearance between the two groups after ERCP, the results of different studies are inconsistent, while there is no significant difference in this study (23, 24, 26, 29–31).

This study has some limitations. First, this study only had 34 choledocholithiasis patients with cirrhosis, the sample size was small. Secondly, because some patients had biliary tract infection before surgery and were treated with antibiotics, these patients were not classified as postoperative biliary tract infection, thus affecting the results. Last, a one-month follow-up period may be insufficient to capture late-onset complications, particularly in cirrhotic patients who may experience a delayed progression of liver dysfunction or other complications related to their underlying condition. Our findings suggest liver cirrhosis may increase the incidence of delayed bleeding and mortality after ERCP for CBDS. However, given the observational nature of this study and the small number of cirrhosis patients, no causal relationships can be inferred. Further prospective studies with larger cohorts are required to confirm these observations and to better understand the underlying mechanisms.

In conclusion, while this study found that liver cirrhosis may increase the incidence of delayed bleeding and mortality after ERCP for CBDS. However, due to the small sample size of this study, a multivariate logistic analysis could not be conducted, thus could not rule out other factors that might be independent of liver cirrhosis. Future research with larger, well-designed studies is necessary to draw more definitive conclusions regarding these associations.

## Data Availability

The raw data supporting the conclusions of this article will be made available by the authors, without undue reservation.
